# Vancomycin AUC-Based Dosing Practices in a Non-Teaching Community Hospital and Associated Outcomes: A One-Year Survey of Uniform Targets for Infections with or without MRSA

**DOI:** 10.3390/pharmacy12010015

**Published:** 2024-01-17

**Authors:** Iftekharul Islam

**Affiliations:** Department of Pharmacy, MedStar Montgomery Medical Center, Olney, MD 20832, USA; iftekharul.z.islam@medstar.net

**Keywords:** vancomycin, AUC, MRSA

## Abstract

Background: Intravenous (IV) vancomycin area under the curve (AUC)-based dosing is used uniformly for Gram-positive organisms in non-teaching community hospitals. However, evidence for using vancomycin AUC-based dosing for non-methicillin-resistant *Staphylococcus aureus* (*non-MRSA*) and less serious infections is limited in the literature. A gap in the literature also exists with respect to comparisons between the outcomes that can be derived using the regimens suggested by Bayesian programs and target doses of the AUC of 400–499 and 500–600. Methods: A retrospective review of all patients hospitalized in a non-teaching community hospital who used AUC-based vancomycin was performed over a 1-year period. Results: Only 17.6% of the included patients had confirmed MRSA. The values for the overall early response rate, 30-day all-cause mortality, and rate of acute kidney injury (AKI) were 50.3%, 11.3%, and 3.8%, respectively, in this population. In regression analysis, compared to non-MRSA infections, a significantly higher rate of early response was seen in patients with MRSA (unadjusted OR = 2.68, 95% CI [1.06–6.76] *p* = 0.04). Patients in the AUC 400–499 group had a non-significant higher incidence of 30 d mortality and new AKI compared to patients in the AUC 500–600 group. In our Kaplan–Meier survival analysis, there was no statistically significant difference between the comparison groups. Conclusions: Early response was lower in patients with non-MRSA compared to patients with MRSA despite achieving the AUC target. There was no apparent difference in clinical outcomes between the higher and lower AUC groups. Further large-scale research is needed to confirm these findings.

## 1. Introduction

The area under the curve/minimum inhibitory concentration (AUC/MIC) is the most accurate way to dose and monitor intravenous (IV) vancomycin for treating MRSA infections. A steady-state AUC/MIC between 400 and 600 ensures optimum outcomes and minimizes the risk of acute kidney injury (AKI) [1,2,3]. The Infectious Disease Society of America (IDSA)/American Society of Health System Pharmacists (ASHP) have updated their guidelines, recommending the use of Bayesian AUC prediction models for all serious MRSA infections (bacteremia, osteomyelitis, pneumonia, meningitis, and endocarditis) based on evidence of superior outcomes derived from this methodology, primarily from studies involving Staphylococcal bacteremia [1]. In AUC-guided vancomycin dosing, random levels for Bayesian modeling can be drawn at any time after the administration of at least one dose [1,4]. On the other hand, in trough-based dosing, vancomycin levels are drawn immediately prior to the next dose at a steady state [5]. If an institution uses AUC-based vancomycin dosing for MRSA infections but uses trough-based dosing for other Gram-positive infections, there exists the risk of incorrect dose adjustments if a random level is interpreted as a trough, and vice versa. The question is, why is research needed in the field of IV vancomycin dosing for non-MRSA organisms when IV vancomycin is generally expected to be used for limited durations in this type of population?

Extended IV vancomycin therapy is continued for patients who do not fall into the above guidelines’ criteria for AUC-based dosing [6]. Additionally, studies on antibiotic de-escalation show that the rate of IV vancomycin streamlining at 72 h is only 52% for large academic centers and possibly lower for smaller community hospitals [7]. Broad-spectrum antibiotics such as IV vancomycin are often not de-escalated due to the nature of infection necessitating longer treatment, as is the case in treatment regimens for bacteremia or endocarditis if patients have a steady response, for the presence of shock, or due to delays in culture results [8]. 

Vancomycin AUC targets associated with good outcomes in less serious infections are also limited. Prior to the updated recommendations, vancomycin dosing for non-serious infections, such as skin and soft tissue infections, followed a lower trough goal [9]. However, pharmacokinetic studies showed a poor association between trough values and AUC values [10,11]. More recently, retrospective studies have shown that an AUC/MIC between 400 and 600 is associated with good early response and a low risk of acute kidney injury for both Enterococcal infections and non-serious infections [12,13]. Moreover, the early attainment of AUC values greater than 389 is associated with lower clinical failure rates in *Enterococcus faecium* bacteremia [14]. However, a gap in the literature exists with respect to the reporting of outcomes associated with infections caused by non-MRSA pathogens such as methicillin-sensitive *Staphylococcus aureus* (MSSA), methicillin-resistant *Staphylococcus epidermidis* (MRSE), and other coagulase-negative *Staphylococcus* spp. since antibiotics are expected to be streamlined once cultures show definitive organism(s) [1].

One additional issue surrounding IV vancomycin AUC-based dosing concerns selecting the appropriate regimen from the many suggestions provided by Bayesian programs for each patient scenario. Vancomycin regimens targeting steady-state AUC values between 500 and 600 are commonly hypothesized by pharmacists in our practice to be better for serious infections compared to regimens with a lower AUC target of between 400 and 499.

The current literature, therefore, does not fully address the following issues related to vancomycin AUC-based dosing in practice: how frequently is IV vancomycin AUC-based dosing continued for patients without MRSA? Are clinical outcomes any different if an AUC target between 400 and 600 is used uniformly for all Gram-positive infections where an extended duration of IV vancomycin treatment is used? Are there any differences in outcomes between MRSA and Gram-positive non-MRSA infections? Does targeting a vancomycin AUC value between 400 and 499 lead to any differences in outcomes compared to targeting a higher AUC value (e.g., between 500 and 600) in a population of patients with both MRSA and non-MRSA?

## 2. Materials and Methods

The primary objective of this retrospective chart review is to describe the proportions of MRSA and non-MRSA infections at our institution in patients who used AUC-based IV vancomycin dosing. The early clinical responses, mortality, and rates of AKI in a cohort of all patients admitted over a period of one year are also described. The secondary exploratory objectives of this paper are to (1) compare efficacy and safety outcomes between MRSA and Gram-positive non-MRSA infections in a population treated uniformly with AUC-based dosing and (2) compare the efficacy and safety outcomes associated with targeting average steady-state AUC values between 400 and 499 and between 500 and 600 in this population. 

### 2.1. Patient Selection

We included all patients in the initial screening, starting one year after the institutional implementation of AUC-based dosing. We did not include patients from the first year of AUC-based dosing implementation due to the large variation in the vancomycin dosing approaches among the pharmacists and due to the potential effects derived from the presence of COVID-19. Using an electronic drug inquiry program, Discern Analytics™ version 2.0, all patients who were administered any formulation of vancomycin in the hospital between 1 May 2022 and 30 April 2023 were identified. Only patients treated with IV vancomycin using AUC-based dosing for at least 72 h during the specified period were selected for our analysis. Patients who were excluded from our analysis included dialysis patients, patients with renal failure, pediatric patients, patients requiring surgical prophylaxis, patients receiving treatment gynecologically, patients with definitive Gram-negative infections, and patients who initiated therapy only for positive blood cultures, which later turned out to be contaminants, as determined by ID specialists. 

### 2.2. Data Collection

For patients with multiple admissions, their regimen with the longest duration of AUC-based vancomycin dosing was selected for review to maximize the possibility of AUC attainment at a steady state. Patient demographic information, pertinent laboratory data, antibiotics, and information on ID consultation were all collected from electronic health records by individual patient chart reviews. 

Vancomycin AUC calculations and predictions were performed using InsightRX™ Nova (version 1.52.2), an integrated clinical decision support tool that uses Bayesian modeling [15]. Average steady-state AUC values were calculated for each patient by taking the average of the predicted steady-state AUC values for each day of AUC-based dosing. At least 1 level up to a maximum of 3 levels was considered to model the AUC achieved after 48 h using Bayesian Software. All AUC/MIC values assumed an MIC of 1.0 in InsightRX™, which is indicated only by the “AUC” values in this article described in milligrams times hour per liter (mg*h/L). The average vancomycin dose for each patient was calculated from all doses administered during therapy. 

The calculation of the minimum inhibitory concentration (MIC) values of vancomycin for the reported organisms was performed using BD Phoenix™, an automated identification and susceptibility testing system that identifies MIC values using broth-based microdilution technology [16].

The clinical response was defined as clinical improvement in at least one or a combination of the following: the resolution of leukocytosis (WBC > 10.8 k/µL per local lab standard), the resolution of moderate-to-high-grade fever (>38 °C) accompanied by an ID specialist or hospitalist’s evaluation of an improvement in the patient’s clinical symptoms, a decrease in vasopressor requirement, a patient-reported improvement in terms of symptoms, radiologic improvement, a decrease in respiratory support, a downgrade to lower-level care, or discharge without significant worsening for any of these. Clinical failure was defined as the worsening of any of the above signs and symptoms accompanied by an ID specialist or hospitalist’s evaluation indicating clinical worsening or death. 

Mortality data were collected using the health information exchange records of hospitals in Maryland, the District of Columbia, and Virginia. The start of vancomycin therapy was defined as day one of vancomycin therapy during the selected admission period. For patients with intermittent periods of therapy in the same admission period (rare), day one was defined as day one of the periods with the longest duration after at least 48 h of the absence of any administration. The time to source control in relation to vancomycin therapy was calculated by taking the time difference between the start of IV vancomycin treatment and the day of any of the following procedures: incision and drainage or abscess drainage or the removal of infected lines and drains. 

AKI was defined as serum creatinine (SCr) with increases of >0.3 mg/dL in 48 h or the doubling of SCr post the start of therapy. Microsoft Excel™ was used to record data. 

### 2.3. Statistical Analyses

All descriptive and inferential statistics were calculated using SPSS Professional Edition version 29. An independent sample T-test was used to compare continuous data, and either Pearson’s chi-squared test or Fisher’s exact test was used where applicable to compare categorical variables. The significance level for the statistical tests was defined as *p* < 0.05 (two-tailed). Levene’s test was used for the equality of variance testing regarding the T-test results. Patients with no culture data were included in the descriptive statistics but not in the regression analysis. Binary logistic regression was used to find any statistical differences in unadjusted mortality, early response, or seven-day response against the independent variables. Multivariate logistic regression, including potential confounders such as baseline creatinine clearance, sepsis diagnosis, diabetes, Gram-negative coverage, and ICU admission, was performed for any significant outcome from the univariate analyses. Kaplan–Meier survival analysis with a log-rank test was used to compare 1-year survival between the groups. 

## 3. Results

### 3.1. Patients

**Figure 1:**
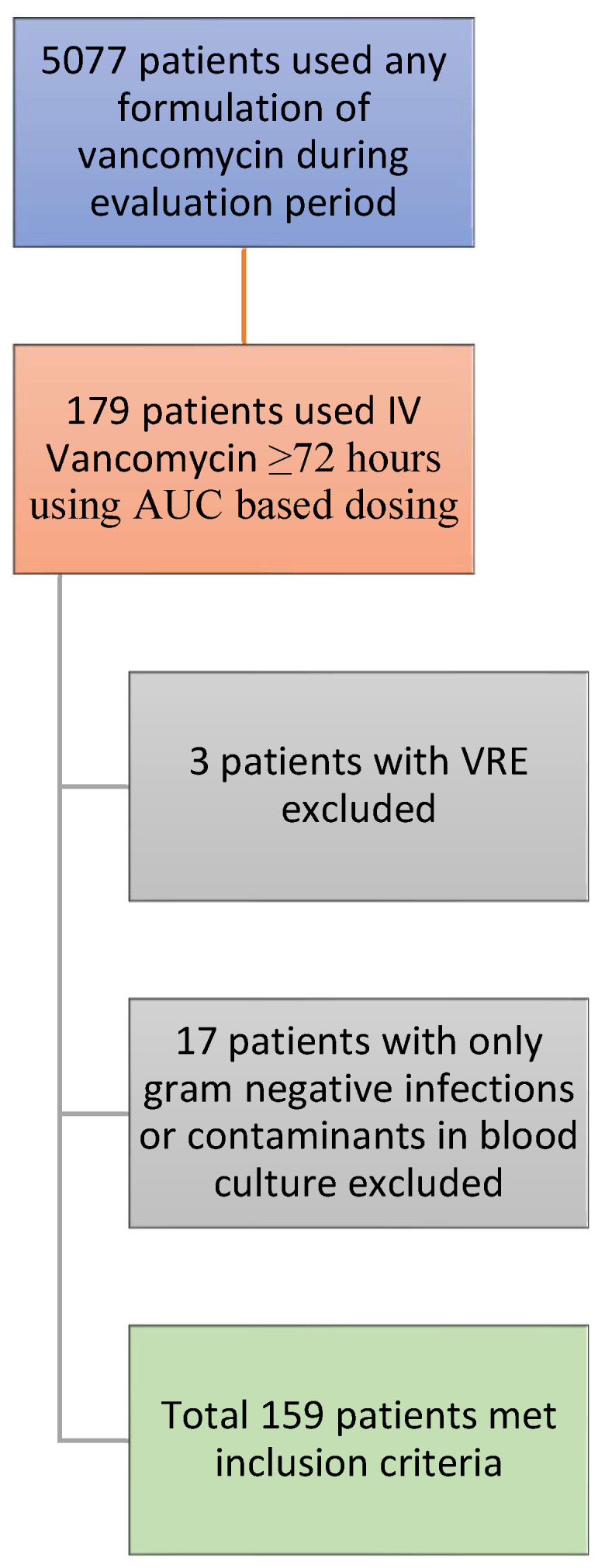
Patient selection.

From 1 May 2022 to 30 April 2023, a total of 5077 patients who received at least one dose of any formulation of vancomycin were identified. After applying the exclusion criteria listed above, 179 patients were found, of which 3 had vancomycin-resistant *Enterococcus* (VRE), and 17 had exclusively Gram-negative infections or contaminants only in blood cultures (Figure 1). Our final sample size was 159 patients. The baseline characteristics of the participants are presented in Table 1. The most common comorbidity was hypertension, and the most common indicators for IV vancomycin treatment were SSTI, osteomyelitis, and pneumonia.

### 3.2. Primary Outcomes

Only 28 of 159 (17.6%) patients grew MRSA in cultures pertinent to the infection. The percentage of patients who met the guidelines’ definition of serious MRSA was only 11.3%. Infections with MRSA and/or *Enterococcus* spp. were found in 28.9% of patients. Polymicrobial infections consisting of Gram-negative and Gram-positive organisms were found in 23.9% of the overall study population. The most common non-MRSA, non-Enterococcal isolates were MRSE, undifferentiated coagulase-negative *Staphylococcus*, and MSSA. The culture results were either negative or unavailable for 46 patients. The details of the MIC distribution of the organisms are listed in Table 2.

The primary clinical outcomes are listed in Table 3. Notably, the values for the overall early response rate, 30-day all-cause mortality, and the rate of AKI were 50.3%, 11.3%, and 3.8%, respectively, for all patients. For poly-microbial infections, the most common pathogens were *Pseudomonas* spp. and *Proteus* spp., and the most used antibiotics were piperacillin–tazobactam and cefepime (Appendix A, Table A1). Source control measures were applied for 61 patients with an average of 3.1 ± 4.2 days relative to the start date of vancomycin treatment. 

### 3.3. Secondary Outcomes

A significantly higher proportion of patients with MRSA had early responses compared to those with non-MRSA. In our regression analysis, compared to non-MRSA infections, MRSA isolates showed higher odds of achieving an early response (unadjusted OR = 2.68, 95% CI [1.06–6.76], *p* = 0.04). After adjusting for baseline creatinine clearance, sepsis diagnosis, the presence of diabetes, appropriate antibiotics for Gram-negative infections, and ICU admission, early response was still significantly better in those with MRSA infections (adjusted OR = 3.36, 95% CI [1.21–9.33], *p* = 0.02). However, there was no statistically significant difference in those without any worsening of symptoms after 7 days (Table 4). Additionally, 5.8% more patients in the non-MRSA group died after 30 days compared to the patients with MRSA, but the lower odds of mortality in the MRSA group were not statistically significant. The rate of acute kidney injury values was similar between the non-MRSA and MRSA groups. Patients in the MRSA group had one less day of hospitalization than those in the non-MRSA group on average, but this result was determined to be statistically insignificant by our regression analysis.

Between patients with serious MRSA and non-serious MRSA, higher percentages of patients in the MRSA group achieved early response and stability at seven days but with a higher 30-day mortality rate and incidence of AKI, although none of these differences were statistically significant. 

Patients with an average steady-state AUC between 400 and 499 had a higher incidence of 30 d mortality and new AKI compared to the patients in the group with an AUC between 500 and 600, but this result was without statistical significance. 

**Figure 2:**
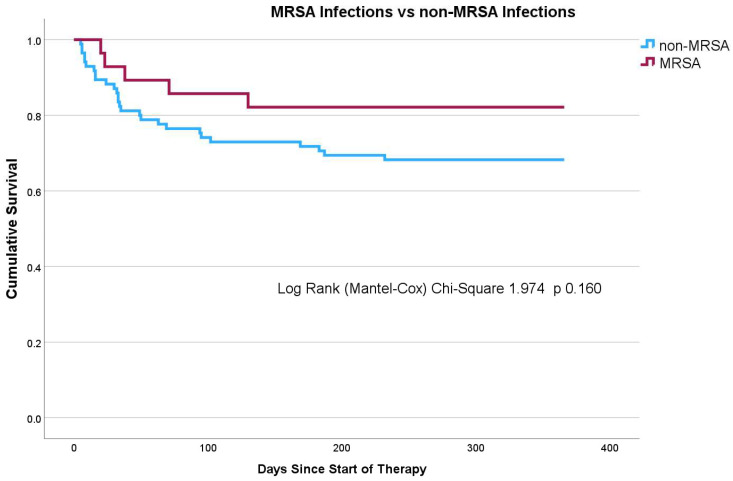
Kaplan–Meier survival curve at one year from therapy initiation.

Finally, in our Kaplan–Meier survival analysis, after using a log-rank test, we observed that there was no statistically significant difference in survival between the comparison groups at one year from therapy initiation, as shown in Figure 2.

## 4. Discussion

In our retrospective review of uniform IV vancomycin AUC-based dosing for all Gram-positive infections, we found that less than one-fifth of all patients had culture-confirmed MRSA. We also determined the overall population’s clinical response, mortality, and acute kidney injury rate values using a uniform AUC target for all Gram-positive infections. All clinical outcomes, except for early response, were similar between patients with and without an MRSA infection. Additionally, there was no significant benefit to targeting an average steady-state AUC above 500 compared to a lower goal of 400–499 in our population. The remainder of this section further discusses the possible implications of each of these findings.

Firstly, our evaluation highlights the need to incorporate clear directions in institutional protocols on vancomycin dosing for Gram-positive non-MRSA or non-serious MRSA infections. A uniform method of dosing vancomycin IV for all Gram-positive infections has the theoretical advantage of operational simplicity and a reduced risk of error in clinical practice. Based on the local prevalence of MRSA, the empiric use of IV vancomycin mostly involves patients without MRSA, as found in our population, which excluded dialysis patients. The low rate of MRSA found in this study was expected based on the local prevalence of the primary types of MRSA. The patients from our community and long-term care facilities are primarily present with community-acquired (CA-MRSA) or healthcare-associated community-onset (HACO-MRSA) infections. The rates of these infections have been low, with those for the year 2020 standing at 4.4 and 12.5 cases per 100,000 patients for CA-MRSA and HACO-MRSA, respectively, according to the Emerging Infections Program Healthcare-Associated Infections (EIP) community report on the incidence of invasive *S. aureus* [17]. On the other hand, the rates of Gram-positive non-MRSA, such as invasive MSSA, were higher than MRSA for both CA-MSSA (12.7) and HACO-MSSA (17.1) per 100,000 patients in EIP areas. Additionally, dialysis patients, who are traditionally excluded from AUC-based dosing, represent many patients with MRSA. The incidence rates in dialysis patients were reported to be 1496 for HACO-MRSA [17]. 

Secondly, IV vancomycin, a broad-spectrum agent that has coverage against both MRSA and Gram-positive non-MRSA organisms, should be primarily reserved for use in treating MRSA for optimum outcomes. In our selected cohort, infections without MRSA had a 2.6 to 3.3 times less likelihood of attaining an early response, although the other long-term outcomes were similar to infections with MRSA. Early response rates for MRSA infections similar to ours have previously been reported in retrospective studies, but studies reporting early response rates for Gram-positive non-MRSA using AUC-based dosing are limited in the literature [13,18]. The present paper is the first study to report a clear difference in early response in Gram-positive non-MRSA, and the results in this regard were found to be significant in the selected cohort. Switching to a non-vancomycin antibiotic may be a better option than continuing with AUC-based vancomycin dosing if MIC is reported to be greater than 1.0. In our population, patients in the non-MRSA group ran the risk of not achieving the stated AUC target due to a substantial proportion of organisms having an MIC of 2.0. An exploratory comparison of the baseline characteristics between the MRSA and non-MRSA groups (presented in Table A2) helped to summarize the other key variables that might directly affect the time to clinical response. 

We also observed a higher non-significant absolute failure rate after seven days in our non-MRSA population (14.1%) compared to the MRSA group (10.7%). Similarly, higher failure rates with Gram-positive non-MRSA isolates were found by Katip, W et al. in a retrospective observational study of 312 patients with Enterococcal infections sensitive to vancomycin. The authors found a failure rate of 15.55% at the end of 9 to 10.5 days of therapy despite the achievement of AUC values greater than or equal to 400 (calculated using the Bayesian program BestDose™) [12]. 

The long-term outcomes regarding 30-day mortality for our patients with MRSA infections (7.1%) are similar compared to those reported in observational studies of patients who used IV vancomycin Bayesian AUC-based dosing for indications not specific to endocarditis (2.6% to 6.2%) [13,19]. However, for MRSA-complicated bacteremia and infective endocarditis, one study reported that crude mortality was as high as 16% even after meeting the AUC goal [20]. Considering the lower rate of bacteremia and a similar rate of endocarditis compared to the MRSA population, mortality was higher (12.9%) for infections with Gram-positive non-MRSA in our population [13,19,20]. In several retrospective studies involving severely ill patients suffering from the *E. faecium* infection, a Gram-positive non-MRSA-associated bacteremia showed no reduction in mortality despite achieving the vancomycin AUC goal [21,22]. One retrospective study with primarily vancomycin-susceptible *Enterococci* showed 17.5% all-cause mortality, while another with *E. faecium* bacteremia had a mortality of 26.7%, despite more than 80% of patients achieving the specified vancomycin AUC target [22,23]. Previous studies have also shown that treatment with anti-Staphylococcal penicillin or cefazolin, after adjusting for confounders, provided a 43% decrease in the risk of mortality compared to IV vancomycin as a definitive therapy for MSSA, another Gram-positive non-MRSA organism, and/or associated bacteremia [18]. Other studies have shown how empiric IV vancomycin treatment streamlined by a median of three days of nafcillin or cefazolin resulted in a 69% decrease in mortality risk compared to those who continued without streamlining [24]. Additionally, a systematic review and meta-analysis showed equivalent clinical cure rates in MSSA bloodstream infections when ceftriaxone was used compared to standard-of-care treatments involving nafcillin, cefazolin, or oxacillin [25]. 

The rates of AKI in our patients with MRSA (3.6%) were higher than the reported rates of AKI in 1 to 1.4% of patients in another study when vancomycin AUC was limited to less than 600 [19]. A higher rate of poly-microbial infections in the MRSA group, which resulted in concomitant piperacillin–tazobactam use, may have contributed to inflating the rate of AKI in our study [26,27,28,29,30]. Interestingly, regarding acute kidney injury rates, in studies involving non-MRSA or non-serious MRSA, significantly higher rates have been reported for patients with non-MRSA compared to patients with MRSA. In fact, nephrotoxicity occurred in 14.96% of patients in a study by Katip et al. when the vancomycin AUC target was greater than or equal to 400 for Enterococcal infections [12]. A higher incidence of nephrotoxicity occurred in a similar portion of patients with CKD (38.46%) and in patients concomitant with using other nephrotoxic drugs such as piperacillin–tazobactam (14.53%) [12]. Additionally, patients with complicated SSTI in Alosaimy et al.’s study cohort had an AKI rate of 10.4%, although 71.4% of the patients with AKI in that study equaled or exceeded the AUC target of 600 [13]. Overall, the evidence in the literature does not support an attributable increased AKI rate in Gram-positive non-MRSA patients compared to MRSA patients when the target AUC value was kept between 400 and 600. Significantly higher rates of vancomycin nephrotoxicity were associated with AUC values greater than 600 in [31,32].

Finally, our results imply that pharmacists may choose any AUC-based regimen that targets an AUC value between 400 and 600 when using a uniform AUC target for Gram-positive organisms. However, discretion should be practiced while choosing a regimen that targets an AUC closer to 400 compared to one between 500 and 600 for serious MRSA infections since we found two-fold non-significant mortality in the lower AUC group. For definitive MRSA infections, the AUC/MIC breakpoint associated with the best outcomes was previously shown to depend on both the severity of the disease and the MIC determination method. A previous study involving patients with complicated MRSA bacteremia and endocarditis had an attributable mortality of 16% when the day-one AUC/MIC, measured using the Epsilometer test (E-test), was greater than or equal to 211 [20]. However, using the broth microdilution method, a day-one AUC greater than 600 was associated with a 2.3-fold lower risk of clinical failure compared to using an AUC less than or equal to 600 in a retrospective study of patients with infective endocarditis [33]. In another retrospective study of patients with MRSA bloodstream infections, day-one AUC/MIC values greater than or equal to 303 and day-two AUC/MIC values greater than or equal to 320 (determined by the E-test) were associated with 30-day mortality rates of 12.3% and 14.1%, respectively [34]. However, in the same study, using the BMD method for the MIC calculation, the daily AUC/MIC breakpoint was recommended to be between 550 and 650 [34]. 

Our review has several limitations. This is a single-center review, so the replicability of the results may be limited by the local prevalence of MRSA. Our results may also have recall bias since most of the data we used were taken retrospectively from patient charts. Delays in culture results often impacted the duration of empiric IV vancomycin use. After applying the exclusion criteria, we found that fewer MRSA patients were encountered during the study period compared to patients with non-MRSA, reducing the precision of the confidence intervals found by our regression analysis. Moreover, this review lacks a power analysis for the secondary outcomes, so any significant result is of low reliability. The descriptive nature of the primary objectives and recent adaptations to the AUC-based dosing of a limited number of patients precluded us from achieving an adequate sample size. A case–control study with 65 patients per group (including early responders and non-responders) would provide 80% power with a 95% confidence level to detect similar odds to those presented here [35]. Nevertheless, one of the primary goals of this review was to evaluate outcomes in a real-world setting where non-MRSA infections are encountered more than MRSA infections. Finally, a large portion of our patients had poly-microbial infections as potential confounders, although the effects derived from the inadequate treatment of these Gram-negatives were potentially minimized since 100% of patients received appropriate treatment for Gram-negative organisms, with >90% receiving an ID consultation.

## 5. Conclusions

In our study cohort, vancomycin Bayesian AUC-based dosing, which continued for greater than or equal to 72 h in our population, included more non-MRSA infections and non-serious infections than MRSA or serious MRSA infections. While our overall results indicate the possibility of better outcomes in patients with MRSA compared to non-MRSA, the only possibly significant difference was the higher likelihood of an early response in the MRSA group. Additionally, we failed to show any significant difference between targeting a steady state AUC between 400 and 499 and 500 and 600, despite a higher incidence of mortality being observed in the lower AUC group. The results of our study are limited by the study’s small sample size and the retrospective nature of our evaluation. Further large-scale prospective research is recommended to confirm our findings for possible application in practice.

## Figures and Tables

**Table 1:** Baseline characteristics of the overall population.

Patient Characteristics N = 159		Mean ± SD or No. (%)
Age (years)		69.3 ± 17.1
Weight (kg)		80.8 ± 25.4
BMI (kg/m^2^)		27.9 ± 8.3
Baseline WBC (10^3^/µL)		11.8 ± 6.2
Baseline temperature (degrees celsius)		37.2 ± 0.8
Baseline CrCl (mL/min)		75.7 ± 47.0
Male Female		84 (52.8) 75 (47.2)
Medical conditions	Hypertension	95 (59.7)
	Dyslipidemia	63 (39.6)
	Chronic kidney disease	56 (35.2)
	Diabetes Mellitus	50 (31.4)
	Cancer	27 (17.0)
	Venous thromboembolism	25 (15.7)
	Cerebrovascular disease	25 (15.7)
	Cardiovascular disease	24 (15.1)
	PAD/PVD	23 (14.5)
	COPD/Asthma	22 (13.8)
	HIV	2 (1.3)
Primary indication	SSTI	51 (32.1)
	Osteomyelitis	41 (25.8)
	Pneumonia	30 (18.9)
	Bacteremia	15 (9.4)
	Abdominal infection	6 (3.8)
	Endocarditis	4 (2.5)
	UTI	4 (2.5)
	Meningitis	2 (1.3)
	Neutropenic fever	2 (1.3)
	Sepsis Empiric	2 (1.3)
	Port infection	1 (0.6)
	Necrotizing fasciitis	1 (0.6)
ICU admission prior to the start of vancomycin		17 (10.7)
Sepsis diagnosis		89 (56.0)
ID consultation		147 (92.5)

Keys: BMI = body mass index, CrCL = creatinine clearance, WBC = white blood cell count, PAD = peripheral arterial disease, PVD = peripheral vascular disease, COPD = chronic obstructive pulmonary disease, HIV = human immunodeficiency virus, SSTI = skin and soft tissue infection, UTI = urinary tract infection; ICU = intensive care unit, ID= infectious disease.

**Table 2:** Microbiology of disease-causing pathogens from patients treated with IV vancomycin.

Organism (s)	Frequency N = 159	MIC (% of Isolates) µg/mL
MRSA	17.6%	0.5 (11.1) 1.0 (88.9)
*Enterococcus* spp.	17.0%	0.5 (11.1) 1.0 (74.1) 2.0 (14.8)
MRSE	13.2%	0.5 (25) 1.0 (65) 2.0 (10)
Coagulase-negative *Staphylococcus* spp. (*undifferentiated*)	12.6%	0.5 (23.5) 1.0 (47.1) 2.0 (29.4)
MSSA	8.2%	1.0 (100)
*Streptococcus* spp.	7.5%	0.25 (33.3) 0.5 (33.3) 1.0 (33.3)
Other Gram-positive organisms	6.9%	1.0 (60) 2.0 (40)
MSSE	2.5%	0.5 (25) 1.0 (50) 2.0 (25)

Key: MIC = minimum inhibitory concentration, MRSE = methicillin resistant *Staphylococcus epidermidis*, MRSA = methicillin resistant *Staphylococcus aureus*, MSSA = methicillin sensitive *Staphylococcus aureus*, MSSE = methicillin sensitive *Staphylococcus epidermidis*.

**Table 3:** Primary clinical outcomes.

Outcome N = 159		Mean ± SD or No. (%)
Vancomycin duration of therapy (days)		8.3 ± 4.5
Average total daily dose (mg/kg/day)		21.3 ± 8.5
Average AUC (mg*h/L)		508.2 ± 43.8
AUC achieved after 48 h (mg*h/L)	400–500	85 (53.5)
	<400	35 (22.0)
	500–600	34 (21.4)
	>600	5 (3.1)
30 day all-cause mortality		18 (11.3)
Early response (72 h)		80 (50.3)
Absence of clinical failure on day seven		142 (89.3)
Acute kidney injury		6 (3.8)
Hospital length of stay (days)		15.6 ± 14.8

Key: AUC = area under the curve at steady state.

**Table 4:** Secondary outcomes.

Outcome	Confirmed MRSA (*n* = 28) vs. Non-MRSA (*n* = 85) OR (95% CI)	Serious MRSA (*n* = 18) vs. non-MRSA or Non-Serious MRSA (*n* = 117) OR (95% CI)	AUC 400–499 (*n* = 66) vs. AUC 500–600 (*n* = 92) OR (95% CI)
Early response rate (72 h)	71.4% vs. 48.2% OR 2.68 (1.06–6.76) p 0.04 aOR 3.36 (1.21–9.33) p 0.02	72.2% vs. 49.6% OR 2.64 (0.89–7.89) p 0.08	45.5% vs. 54.3% OR 1.43 (0.76–2.69) p 0.27
Absence of clinical failure on day seven	96.4% vs. 85.9% OR 4.44 (0.55–35.78) p 0.16	94.4% vs. 88.9% OR 2.12 (0.26–17.31) p 0.48	87.9% vs. 90.2% OR 1.27 (0.46–3.49) p 0.64
30-day mortality rate	7.1% vs. 12.9% OR 0.52 (0.11–2.49) p 0.41	11.1% vs. 9.4% OR 1.20 (0.24–5.94) p 0.82	15.2% vs. 8.7% OR 0.53(0.20–1.43) p 0.21
Incidence of new AKI	3.6% vs. 3.5% OR 1.01 (0.10 to 10.14) p 0.99	5.6% vs. 2.6% OR 2.23(0.22–22.74) p 0.50	4.5% vs. 3.3% OR 0.71 (0.14–3.62) p 0.68
Duration of hospital stay (days)	15.29 vs. 16.35 MD −1.07 (−7.74 to 5.61) p 0.75	16.39 vs. 15.35 MD 1.04 (−6.23–8.31) p 0.78	16.01 vs. 15.15 MD 0.86 (−3.89–5.61) p 0.72

Keys: OR = odds ratio, aOR = adjusted odds ratio, CI = confidence interval, MD = mean difference.

## Data Availability

Data are unavailable due to privacy rules.

## Appendix A

**Table A1:** Gram-negative organisms in polymicrobial infections and other antibiotics used.

Gram Negative Organism (N = 38)	No. of Patients
*Pseudomonas* spp.	14
*Proteus* spp.	13
*Klebsiella* spp.	8
*Escherichia coli*	8
Other Gram-negative organisms	5
*Enterobacter* spp.	5
*Morganella* spp.	4
*Citrobacter* spp.	3
*Acinetobacter* spp.	3
*Providencia* spp.	2
Additional IV Antibiotics Used (N = 159)	
Cefepime	28
Piperacillin-tazobactam	26
Ceftriaxone	17
Other antibiotics	17
Meropenem	11
Aztreonam	3
Levofloxacin	3
Ampicillin-Sulbactam	2
Gentamicin	1
Colistin	1

**Table A2:** Baseline characteristics of patients with MRSA vs. non-MRSA.

Patient Characteristics	MRSA N = 28 No. (%) or Mean ± SD	Non-MRSA N = 85 No. (%) or Mean ± SD	Significance (P)
Female gender	12 (42.9)	37 (43.5)	0.95
Age (years)	67.8 ± 14.2	68.8 ± 17.8	0.78
Weight (kg)	84.7 ± 26.1	78.4 ± 24.2	0.23
BMI (kg/m^2^)	29.1 ± 8.4	26.9 ± 7.7	0.21
Baseline WBC (10^3^/µL)	12.4 ± 6.5	11.6 ± 6.6	0.56
Baseline temperature (degrees celsius)	37.1 ± 1.1	37.2 ± 0.7	0.40
Baseline CrCL (mL/min)	81.1 ± 69.8	76.3 ± 39.6	0.65
Average AUC (mg*h/L)	526.5 ± 82.3	504.7 ± 30.7	0.18
Vancomycin duration (days)	11.5 ± 7.0	7.9 ± 3.9	0.01
Vancomycin dose (mg/kg/day)	19.4 ± 6.9	22.2 ± 8.4	0.12
Time to source control (days)	3.4 ± 4.2	2.3 ± 2.4	0.24
Medical conditions			
Hypertension	15 (53.6)	48 (56.5)	0.79
Diabetes Mellitus	15 (53.6)	23 (27.1)	0.01
Dyslipidemia	11 (39.3)	31 (36.5)	0.79
Chronic kidney disease	11 (39.3)	27 (31.8)	0.46
Cerebrovascular disease	6 (21.4)	11 (12.9)	0.36
PAD/PVD	5 (17.8)	8 (9.4)	0.30
Cardiovascular disease	4 (14.3)	9 (10.6)	0.73
Venous thromboembolism	4 (14.3)	16 (18.8)	0.78
COPD/Asthma	4 (14.3)	11 (12.9)	>0.99
Cancer	2 (7.1)	19 (22.4)	0.07
HIV	1 (3.6)	1 (1.2)	>0.99
AUC after 48 h (mg*h/L)			
<400	6 (21.4)	19 (22.4)	
>600	1 (3.6)	3 (3.5)	
400–499	15 (53.6)	46 (54.1)	
500–600	6 (21.4)	17 (20)	
Polymicrobial with Gram-negatives	13 (46.4)	25 (29.4)	0.10
ICU admission prior to initiation	6 (21.4)	9 (10.6)	0.20
Sepsis diagnosis	16 (57.1)	48 (56.5)	0.95
ID consultation	26 (92.9)	83 (97.6)	0.26
Primary indication			
SSTI	10 (35.7)	22 (25.9)	
Osteomyelitis	9 (32.1)	26 (30.6)	
Bacteremia	5 (17.9)	10 (11.8)	
Pneumonia	3 (10.7)	10 (11.8)	
Endocarditis	1 (3.6)	3 (3.5)	
Meningitis	0	1 (1.2)	
Necrotizing fasciitis	0	1 (1.2)	
Neutropenic fever	0	2 (2.4)	
Abdominal Infection	0	3 (3.5)	
Port infection	0	1 (1.2)	
Sepsis Empiric	0	2 (2.4)	
UTI	0	4 (4.7)	
